# Characterisation of a secreted MFSD6-Fc microbody as a decoy receptor for respiratory enterovirus D68

**DOI:** 10.1016/j.ebiom.2025.105915

**Published:** 2025-09-08

**Authors:** Zhaoxue Li, Huili Li, Xize Liu, Junfeng Zhou, Delong Gao, Wanying Yang, Huiming Xia, Chao Dou, Zhenglei Yu, Haoran Guo, Wei Wei

**Affiliations:** a Cancer Centre, The First Hospital of Jilin University, Changchun, Jilin, 130021, China; b Institute of Virology and AIDS Research, The First Hospital of Jilin University, Changchun, Jilin, 130021, China; c Department of Dermatology, First Hospital, Jilin University, Changchun, Jilin, 130021, China; d The Key Laboratory of Bionic Engineering, Ministry of Education, Jilin University, Changchun, Jilin, 130012, China; e Antibiotics Research and Re-evaluation Key Laboratory of Sichuan Province, Sichuan Industrial Institute of Antibiotics, School of Pharmacy, Chengdu University, Chengdu, 610106, China; f Institute of Translational Medicine, Key Laboratory of Organ Regeneration and Transplantation of Ministry of Education, The First Hospital of Jilin University, Changchun, Jilin, 130021, China

**Keywords:** EV-D68, MFSD6, Virus receptor, Antiviral agent, Decoy receptor

## Abstract

**Background:**

Enterovirus D68 (EV-D68) is a prominent non-polio enterovirus known to cause severe respiratory infections and poliomyelitis-like illnesses in children. Recently, we identified MFSD6 as a receptor for EV-D68, providing a potential target for blocking viral entry into cells. This study aimed to develop an MFSD6-based decoy receptor to neutralise EV-D68 and elucidate its mechanism of action.

**Methods:**

In this study, we engineered a secreted MFSD6-Fc microbody (secMFSD6 Mb) and evaluated its efficacy using in vitro binding assays (co-immunoprecipitation, RT-qPCR), electron microscopy, and functional studies in EV-D68-infected respiratory cell lines (Calu-3, BEAS-2B, A549), primary human bronchial epithelial cells (HBECs), and a neonatal ICR mouse model (n = 9 per group) infected with EV-D68. Statistical significance was determined by two-way ANOVA and t-test (GraphPad Prism 8.0.2; significance threshold P < 0.05).

**Findings:**

secMFSD6 Mb occupies the receptor-binding sites on the viral surface, reducing virus attachment to cells by >90% (n = 3 biological replicates). Electron microscopy showed conversion of intact virions to empty capsids after Mb treatment, and sucrose-gradient analysis demonstrated a 6-fold increase in free viral RNA (F2 fraction) compared with control. In mice challenged with 1 × 10^7^ TCID_50_ of US/MO/14-18947, secMFSD6 Mb increased 15-day survival from 11% (1/9) to 89% (8/9).

**Interpretation:**

This decoy receptor strategy may support the development of effective therapeutic approaches against EV-D68 infection.

**Funding:**

HYPNSFC Excellent Young Scientist Fund (32222005), the 10.13039/501100001809National Natural Science Foundation of China (82372226, 82172246), the National Major Project for Infectious Disease Control and Prevention (2018ZX10731-101-001-016).

**Research in context**

**Evidence before this study**

Enterovirus D68 (EV-D68) is a non-polio enterovirus that has emerged as a significant cause of severe respiratory infections and neurological complications, particularly in children. Previous studies have identified several cellular receptors for EV-D68, including sialic acid, ICAM-5, and heparan sulphate. Recently, MFSD6 was identified as a key receptor for EV-D68 in respiratory cells. Despite these advancements, there remains a lack of effective therapeutic interventions for EV-D68 infections.

**Added value of this study**

Our study introduces a therapeutic strategy by engineering a secreted MFSD6 microbody (secMFSD6 Mb) that acts as a decoy receptor to specifically recognise and inhibit EV-D68. This microbody not only blocks the virus from attaching to host cells but also directly disrupts the structural integrity of the virus, leading to inhibition of viral replication in both in vitro and in vivo models. The dual mechanism of action-receptor competition and virion destabilization could represents a potential advancement over conventional antiviral approaches. Our findings demonstrate antiviral activity against diverse EV-D68 strains, including historical and contemporary epidemic isolates, highlighting the potential for this strategy to address the evolving nature of EV-D68.

**Implications of all the available evidence**

Combining our results with existing evidence underscores the critical role of MFSD6 in EV-D68 infection and establishes the decoy receptor approach as a promising therapeutic avenue. This strategy could potentially be extended to other viruses that exploit similar entry mechanisms. For human health, our findings provide a foundation for developing effective treatments to prevent severe respiratory illnesses and neurological complications associated with EV-D68. Future research should focus on optimising the MFSD6 microbody for clinical application, including evaluating its long-term safety and efficacy in larger animal models and eventually in human trials. Additionally, further exploration of the interaction between MFSD6 and EV-D68 could enhance our understanding of viral entry mechanisms and inform the design of next-generation antiviral agents.

## Introduction

Enterovirus D68 (EV-D68) is a member of the species *Enterovirus* D in the genus *Enterovirus*, family *Picornaviridae*. Initially identified in 1962, EV-D68 was long regarded as a rare pathogen until a significant outbreak occurred in North America in 2014, leading to its emergence as a serious global public health concern.^1^, ^2^, ^3^, ^4^, ^5^ EV-D68 possesses unique biological characteristics among enteroviruses: unlike typical enteroviruses such as poliovirus, EV-A71, and echoviruses, which are primarily transmitted via the fecal-oral route, EV-D68 is mainly spread through respiratory pathways.^6^, ^7^, ^8^, ^9^, ^10^, ^11^, ^12^ The virus primarily causes respiratory illnesses; however, with the increasing number of cases, its association with acute flaccid myelitis (AFM), a neurological condition resembling poliomyelitis, has garnered heightened attention.^13^, ^14^, ^15^, ^16^, ^17^, ^18^, ^19^ Currently, there is a lack of widely effective clinical interventions against EV-D68 infections, making it crucial to investigate the mechanisms of viral pathogenesis and transmission in order to identify potential therapeutic targets.

EV-D68 is a single-stranded positive-sense RNA virus with a genome that contains a single open reading frame (ORF) encoding a precursor protein. This precursor protein is cleaved by proteases to produce four structural proteins (VP1-VP4) and seven non-structural proteins (2A-2C, 3A-3D).^20^, ^21^, ^22^, ^23^, ^24^, ^25^ Notably, an open reading frame (ORF2/uORF) that we and others previously discovered in the enterovirus family is absent in the EV-D68 genome. Given that the protein encoded by ORF2 (ORF2p/Up) plays a significant role in the infection of human intestinal epithelial cells by enteroviruses, the lack of ORF2p in EV-D68 may explain its inability to spread via the fecal-oral route.^6^^,^^26^

The lifecycle of EV-D68 can be divided into several crucial steps: virus adhesion and invasion, viral RNA replication and protein synthesis, viral particle assembly, and virus release.^7^^,^^27^, ^28^, ^29^ Among these, the entry of the virus into host cells is a critical initial step, dependent on the mediation of specific receptor molecules on the surface of host cells.^30^ Over the years, sialic acid, ICAM-5, and heparan sulphate have been identified as cellular receptors for EV-D68.^31^^,^^32^ Recently, our laboratory identified the membrane protein MFSD6 as a key receptor for EV-D68 infection in respiratory cells.^33^ The identification of MFSD6 as EV-D68 receptor not only elucidates the molecular mechanisms involved in viral invasion of host cells but also provides a theoretical foundation for developing targeted therapeutic strategies to block viral entry.^34^

In this study, we successfully designed and constructed a stable, secreted MFSD6 microbody that can specifically recognise EV-D68 viral particles. By occupying the receptor-binding epitopes on the viral particle surface, the MFSD6 microbody effectively inhibits the binding of EV-D68 to the MFSD6 receptors on target cells. Further biochemical analyses and electron microscopy observations revealed that the MFSD6 microbody can directly disrupt the structural integrity of EV-D68 viral particles, demonstrating inhibition of viral replication in both in vitro and in vivo experiments. This innovative intervention strategy exhibits potential for broad applicability, suggesting utility for the development of therapeutic approaches against EV-D68 infections.

## Methods

### Study design

This is a pre-clinical, proof-of-concept study combining in vitro and in vivo approaches to evaluate the antiviral activity of a secreted MFSD6-Fc microbody (secMFSD6 Mb) against EV-D68. The experimental workflow comprised four sequential modules: (i) biochemical characterisation of secMFSD6 Mb binding to diverse EV-D68 strains; (ii) cell-based assays measuring attachment, entry, replication and cytopathic effects in human respiratory cell lines and primary HBECs; (iii) ultracentrifugation and electron microscopy analyses to assess virion integrity; and (iv) a neonatal mouse survival study to test in vivo efficacy. All assays included appropriate vehicle or control-microbody (secCtl Mb) groups, and statistical analyses were pre-specified.

### Plasmids and reagents

VR1012-MFSD6-HA, VR1012-secCtl Mb, and VR1012-secMFSD6 Mb (sequences are provided in Supplementary Fig. S1) were synthesised and constructed by Generay Biotech Co., Ltd. (Shanghai, China). The following reagents were used: anti-beta tubulin antibody (M1305-2, HuaBio), anti-beta actin antibody (EM21002, HuaBio), anti-EV-D68 VP1 antibody (GTX132313, GeneTex), anti-haemagglutinin (HA) monoclonal antibody (H9658, Sigma–Aldrich), anti-HA Affinity Matrix (11815016001, Roche), and Dynabeads protein G immunoprecipitation kit (10007D, Thermo Fisher Scientific).

For microbody production and purification, HEK293T cells were seeded in 10-cm dishes and then transfected with 10 μg of plasmid encoding secMFSD6-Fc or secCtl-Fc. After 72 h, the supernatant was collected, clarified by centrifugation (12,000×*g*, 20 min, 4 °C), and subjected to immunoprecipitation using Dynabeads Protein G (Thermo Fisher Scientific, 10007D). The purity and yield of secMFSD6-Fc and secCtl-Fc were assessed by Coomassie Brilliant Blue staining and immunoblotting.

### Cells

Human lung carcinoma A549 cells (CRM-CCL-185, ATCC), human lung epithelial Calu-3 cells (HTB-55, ATCC), human bronchial epithelial BEAS-2B cells (CRL-3588, ATCC), human rhabdomyosarcoma RD cells (CCL-136, ATCC), human embryonic kidney 293T cells (CRL-3216, ATCC) were cultured in Dulbecco’s modified Eagle’s medium (DMEM; PM150210, Pricella) supplemented with 10% foetal bovine serum (FBS) and penicillin/streptomycin solution. Primary HBECs isolated from nonsmoking healthy donors and commercially available from ScienCell (3210) were cultured in airway epithelial cell basal medium (PCS-300-030, ATCC) supplemented with the components of a bronchial epithelial cell growth kit (PCS-300-040, ATCC). All cultured cells were maintained at 37 °C in a humidified atmosphere containing 5% CO_2_. Mycoplasma contamination in cell cultures is routinely detected using PCR to ensure their absence.

### Virus

EV-D68 prototype Fermon (VR-1826, ATCC), EV-D68 circulating strains from the 2014 U.S. outbreak, US/MO/14-18947 (VR-1823, ATCC) and US/KY/14-18953 (VR-1825, ATCC) were propagated in RD cells. EV-D68 viruses in the supernatant of infected RD cells were harvested. After three cycles of freezing and thawing, the supernatants were clarified via centrifugation (3000 rpm, 10 min) and passed through a 0.45-mm filter. Viral particles were pelleted through a 20% sucrose cushion in an SW41 Ti rotor at 28,000 rpm for 2 h. Purified virions were stored at −80 °C.

### Viral titre assay

Viral titres were determined by the appearance of CPEs in RD cells via microtitration analysis according to the Reed–Muench method. Briefly, RD cells were cultured under standard conditions in 96-well plates at a density of 10,000 cells/well. EV-D68 was serially diluted (10-fold) with DMEM containing 2% FBS and added to the cells. Viral titres are expressed as the 50% tissue culture infectious dose (TCID_50_).

### Immunoblotting

The cells were harvested and washed twice with cold PBS, lysed in radioimmunoprecipitation assay (RIPA) buffer (1 M Tris 7.8, 1 M NaCl, 1% NP-40, 0.5 M EDTA) at 4 °C for 30 min. The samples were mixed with 1 × loading buffer (0.08 M Tris, pH 6.8, with 2.0% SDS, 10% glycerol, 0.1 M DTT, and 0.2% bromophenol blue) and boiled at 100 °C for 10 min. The samples were subsequently centrifuged at 13,000 rpm for 10 min. The cell lysates were separated via SDS‒PAGE and transferred to nitrocellulose membranes via a semidry apparatus (Bio‒Rad). The membranes were blocked with 5% nonfat powdered milk for 20 min. Then, the nitrocellulose membranes were incubated with various primary antibodies against the proteins of interest; the secondary antibodies used were standard alkaline phosphatase-conjugated anti-rabbit IgG or anti-mouse IgG secondary antibodies (goat anti-rabbit IgG [Jackson ImmunoResearch Laboratories, code: 111-035-045]; goat anti-mouse IgG [Jackson ImmunoResearch Laboratories, code: 115-055-062]). Staining was conducted with 5-bromo-4-chloro-3-indolyl phosphate and nitrotetrazolium blue chloride solutions prepared from chemicals obtained from Sigma–Aldrich.

### Transfection and coimmunoprecipitation (co-IP)

DNA transfection was carried out via polyethylenimine (24765; Polysciences) according to the manufacturer’s instructions. HEK293T cells were harvested at 48 h after transfection, washed twice with cold PBS, lysed in lysis buffer (150 mM Tris, pH 7.5, with 150 mM NaCl, 1% Triton X-100, and complete protease inhibitor cocktail tablets [Roche]) at 4 °C for 30 min, and then centrifuged at 10,000×*g* for 30 min. For HA tag immunoprecipitation, precleared cell lysates were mixed with anti-HA Affinity Matrix, as well as secCtl Mb or secMFSD6 Mb, incubated at 4 °C overnight. The samples were then washed six times with washing buffer (20 mM Tris, pH 7.5; 100 mM NaCl; 0.1 mM EDTA; and 0.05% Tween 20). The beads were eluted with elution buffer (0.1 M glycine-HCl, pH 2.0). The eluted materials were then analysed by SDS‒PAGE and immunoblotting with the appropriate antibodies as previously described.

### Quantitative real-time quantitative PCR (RT-qPCR)

TRIzol reagent (15596026, Thermo Fisher Scientific) was used to extract total RNA from the samples according to the manufacturer’s instructions. The total RNA was then reverse transcribed into cDNA via Hifair III 1st Strand cDNA Synthesis SuperMix for qPCR (11141ES60, Yeasen Biotech). PCR amplification was carried out on a SLAN-96S real-time PCR system (Hongshi, China) with Hieff qPCR SYBR Green MasterMix (11201ES03, Yeasen Biotech). The PCR cycling conditions used were 95 °C for 5 min, followed by 40 cycles of 95 °C for 10 s, 60 °C for 20 s, and 72 °C for 20 s. Single peaks in the melting curve analysis indicated specific amplicons. RT-qPCR was performed with the following primer sequences: GAPDH forward primer, 5'-CCCACTCCTCCACCTTTGACG-3'; GAPDH reverse primer, 5'-CACCACCCTGTTGCTGTAGCCA-3'; EV-D68 forward primer, 5'-ACATGGTGTGAAGAGTCTATTGAGCT-3'; EV-D68 reverse primer, 5'-CCAAAGTAGTCGGTTCCGC-3'. The relative mRNA expression levels of the selected genes were calculated via the 2^−ΔΔCt^ method. GAPDH mRNA was used as an internal control.

### EV-D68 and MFSD6 binding assay

Purified EV-D68 viruses were mixed with secCtl Mb or secMFSD6 Mb at 4 °C for 1 h. Subsequently, 100 μL of protein G agarose was mixed with each sample. After 24 h, the samples were washed six times with wash buffer (20 mM Tris, pH 7.5; 100 mM NaCl; 0.1 mM EDTA; and 0.05% Tween-20). The protein G agarose was eluted with elution buffer (0.1 M glycine-HCl, pH 2.0). The RNA from the samples was extracted for RT-qPCR analysis via EV-D68-specific primers to detect captured EV-D68 vRNA.

### Viral attachment and entry assay

The cells were washed with cold (4 °C) or prewarmed (37 °C) phosphate-buffered saline (PBS) and then incubated with EV-D68 (MOI = 2.0). After incubation at 4 °C or 37 °C for 2 h, the cells were washed two times with cold or prewarmed PBS. Total RNA was extracted, and the relative vRNA level was determined via RT-qPCR, as described above.

### Ethics for animal experiments

All the animal studies were conducted in accordance with Animal Research: Reporting of In Vivo Experiments (ARRIVE) guidelines under protocols approved by the Institutional Animal Care and Use Committee at The First Hospital of Jilin University (approval no. 20250314-01).^35^ Mice were housed under specific pathogen-free conditions in a temperature controlled environment with a 12 h: 12 h light: dark cycle, with 50% humidity and ad libitum access to water and standard laboratory chow. Virus inoculations were performed under anaesthesia with isoflurane, and all efforts were made to minimize animal suffering. In vivo studies were not blinded, and animals were randomly assigned to secMFSD6 Mb/secCtl Mb groups.

### EV-D68 infection in neonatal mice

After incubating secMFSD6 Mb (15 μg) or secCtl Mb with the viral inoculum (1.0 × 10^7^ TCID_50_ of EV-D68 US/MO/14-18947) at room temperature for 30 min, the mixture was administered intraperitoneally to nine one-day-old ICR mice per group were obtained from Beijing Vital River Laboratory Animal Technology Co., Ltd. Body weight and survival were monitored daily. The follow-up interval started at the time of intraperitoneal virus challenge (day 0) and ended at day 15 or death. Mice that survived to day 15 were censored at this time-point.

### Statistics

Statistical data were analysed using GraphPad Prism 8.0.2. For two-sample comparisons, normality (Shapiro–Wilk) and homogeneity of variance (F-test) were assessed; data meeting both assumptions were analysed by unpaired two-tailed t-test, otherwise Welch’s t-test was applied. Multi-factor comparisons employed two-way ANOVA followed by Sidak’s post-hoc test. Survival was analysed using Kaplan–Meier curves, the follow-up interval started at the time of intraperitoneal virus challenge (day 0) and ended at day 15 or death. Exact dependent variables, groups compared, sample sizes (n = 3–9 biological replicates or animals per group), and P values are reported in figure legends. No formal power calculation was performed; the sample size was determined based on previous studies in the literature that assessed similar experimental conditions and outcomes, and was deemed sufficient for detecting meaningful differences given the nature of the experiments. All observations were biologically independent (separately passaged cells or individually housed pups) and treatments were blinded. A P < 0.05 was considered significant. ∗P < 0.05, ∗∗P < 0.01, ∗∗∗P < 0.001, ∗∗∗∗P < 0.0001 and “ns” represents not significant.

### Role of funders

Funders had no role in study design, data collection, data analysis, interpretation, or writing.

## Results

### Engineered secreted MFSD6 microbody binds to EV-D68

**Fig. 1:**
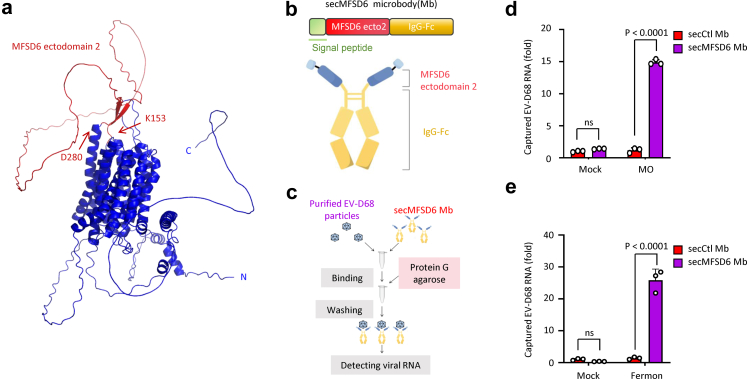
**Engineered secreted MFSD6 microbody binds to EV-D68.** (a) Spatial structure prediction of the MFSD6 protein. The second extracellular domain of MFSD6 is highlighted in red. (b) Schematic diagram of the secMFSD6 microbody (Mb). (c) Schematic illustration of the virion capture assay used to detect the interaction between purified EV-D68 particles and secMFSD6 Mb. (d and e) Purified circulating MO strains (d) or prototype Fermon strains (e) of EV-D68 were incubated with secCtl Mb or secMFSD6 Mb at 4 °C for 1 h. Subsequently, 100 μL of protein G agarose was added and mixed with each sample for 24 h. After washing the samples six times with wash buffer, RNA was extracted for RT-qPCR to detect captured EV-D68 RNA. Data are represented as the mean ± standard deviation (SD) (n = 3). *N* = 3 biological replicates. Two-way ANOVA (d and e) was used to assess statistical significance.

In our previous study to identify MFSD6 as the receptor for EV-D68, we confirmed that the second extracellular domain of MFSD6 (K153-D280) contributes to the interaction between MFSD6 and the virus particle (Fig. 1a). Based on these findings, we designed and upgraded an MFSD6-based antiviral strategy in this study. We engineered a secreted MFSD6 microbody (termed secMFSD6 Mb), which comprises the extracellular domain (K153-D280) of MFSD6, an Fc fragment at the C-terminus, and a signal peptide at the N-terminus to promote the secretion of the translation product from the expressing cells (Fig. 1b, Supplementary Fig. S1).

To assess the biological activity of the engineered secMFSD6 Mb, we performed an in vitro binding assay to evaluate its ability to recognise and capture EV-D68 viral particles. We utilised Protein G agarose to bind the Fc fragment of secMFSD6 Mb and detected the enrichment of EV-D68 virus by measuring the viral RNA levels via RT-PCR (Fig. 1c). Compared with the secreted control microbody lacking the MFSD6 ectodomain (secCtl Mb), secMFSD6 Mb effectively enriched EV-D68 viral particles, including the circulating strain of EV-D68 epidemic virus [US/MO/14-18947 (MO)] (Fig. 1d) and the prototype EV-D68 strain Fermon isolated in 1962 (Fig. 1e). These results suggest that secMFSD6 microbody is biologically active and specifically binds to EV-D68 particles.

### SecMFSD6 Mb interferes with receptor recognition by EV-D68

**Fig. 2:**
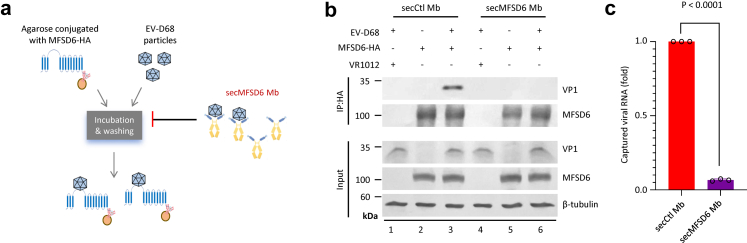
**secMFSD6 Mb inhibits receptor recognition by EV-D68.** (a)The diagram shows that the binding of EV-D68 to secMFSD6 Mb hinders the binding of virus particles to MFSD6. (b) Co-immunoprecipitation (Co-IP) of MFSD6 with EV-D68 MO viruses in the presence of secCtl Mb or secMFSD6 Mb. (c) Captured viral RNA was detected by RT-qPCR. Data are represented as the mean ± SD (n = 3). *N* = 3 biological replicates. Welch’s t-test was used to assess statistical significance.

Subsequently, we investigated the influence of secMFSD6 Mb on the interaction between EV-D68 and its receptor MFSD6 using immunoprecipitation (Fig. 2a). For the immunoprecipitated products, we detected the levels of the viral structural protein VP1 by Western blotting and the viral RNA enrichment by RT-PCR (Fig. 2b and c). Both methods consistently showed that the interaction between EV-D68 and MFSD6 was reduced in the presence of secMFSD6 Mb, indicating that secMFSD6 Mb has the ability to block the recognition of EV-D68 by its receptor (Fig. 2b and c).

**Fig. 3:**
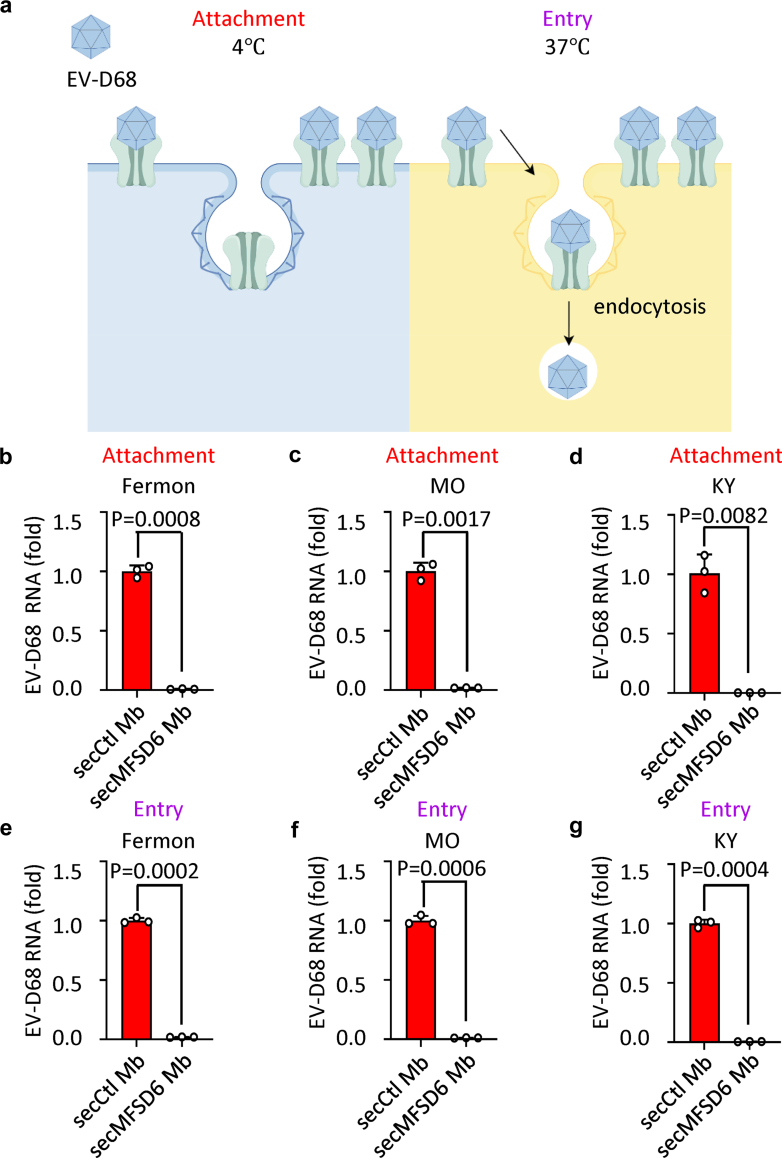
**secMFSD6 Mb inhibits the attachment and entry process of EV-D68.** (a) Schematic of the viral attachment and entry assay. (b–g) EV-D68 viruses were incubated with secCtl Mb or secMFSD6 Mb for 30 min. (b–d) A549 cells were washed with cold PBS before infection and then incubated with treated EV-D68 (Fermon, b; MO, c; KY, d) or secCtl Mb or secMFSD6 Mb at 4 °C for 2 h to facilitate virus attachment. (e–g) A549 cells were washed with prewarmed PBS before infection and then incubated with treated EV-D68 (Fermon, e; MO, f; KY, g) or secCtl Mb or secMFSD6 Mb at 37 °C for 2 h to facilitate virus entry. Data are represented as the mean ± SD (n = 3). *N* = 3 biological replicates. Welch’s t-test (b–g) was used to assess statistical significance.

As many viral receptors are involved in virus attachment to the host cell surface, we further examined whether secMFSD6 Mb could interrupt the virus attachment process. We employed well-established viral attachment and entry assays (Fig. 3a) and found that the ability of all three tested strains of EV-D68 (Fermon, MO, and KY) to attach to host cells was decreased in the presence of secMFSD6 Mb treatment compared with secCtl Mb treatment (Fig. 3b–d). Moreover, secMFSD6 Mb treatment also efficiently inhibited the invasion of EV-D68 viral particles into host cells (Fig. 3e–g). We subsequently confirmed that the inhibitory effects of secMFSD6 Mb on EV-D68 attachment and entry are cell-type independent (Supplementary Fig. S2).

### SecMFSD6 Mb triggers abnormal uncoating of EV-D68

**Fig. 4:**
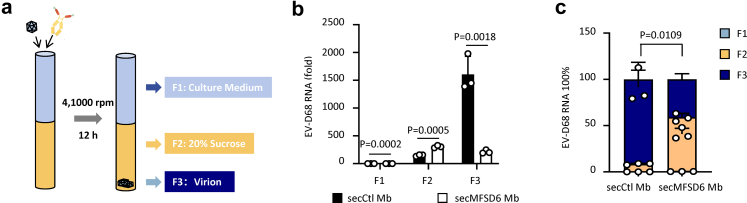
**secMFSD6 Mb destroys the integrity of EV-D68 viral particles.** (a) EV-D68 Fermon viruses were incubated with secMFSD6 Mb or secCtl Mb for 1 h. The samples were then subjected to ultracentrifugation through a 20% sucrose cushion at 41,000 rpm for 12 h at 4 °C. Subsequently, three distinct fractions were collected as indicated. (b) RT-qPCR was used to detect the presence of EV-D68 RNA within the three collected fractions, labelled as F1, F2, and F3. (c) The relative proportions of the F1, F2, and F3 fractions, as determined in (b), were quantified and compared. Data are represented as the mean ± SD (n = 3). *N* = 3 biological replicates. Unpaired t-test (b and c) was used to assess statistical significance.

We previously revealed that the transmembrane protein MFSD6 not only binds to the virus but also catalyses the uncoating process in EV-D68. To determine whether the interaction with secMFSD6 Mb also prompts the uncoating of EV-D68, we employed a viral RNA flotation assay to monitor the release of EV-D68 RNA from viral particles (Fig. 4a). Under normal conditions, intact viral particles can cross a sucrose cushion through ultracentrifugation and mainly accumulate in the F3 fraction (Fig. 4b and c). However, in the presence of secMFSD6 Mb, a dramatic increase in viral RNA levels was detected in the sucrose fraction F2 compared with the F3 fraction (Fig. 4b and c), indicating that secMFSD6 Mb disrupted the integrity of the virus particles.

**Fig. 5:**
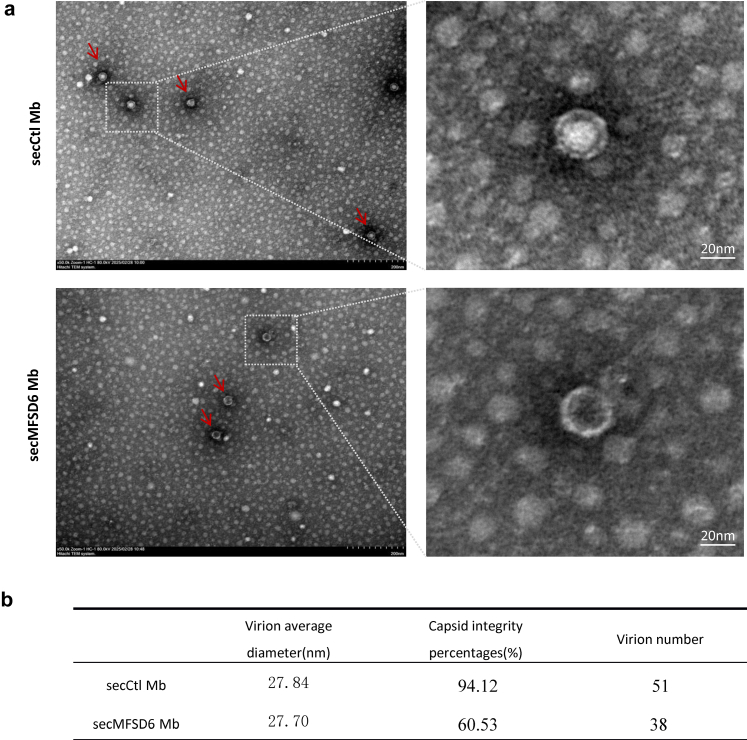
**Electron microscopy observation of the effect of secMFSD6 Mb on the integrity of EV-D68 viral particles.** (a and b) EV-D68 MO viruses were incubated with secCtl Mb or secMFSD6 Mb for 12 h. Virus particles were observed by transmission electron microscopy (TEM). Scale bars represent 200 nm or 20 nm.

To further validate this finding, we used electron microscopy analysis to examine the morphological changes of EV-D68 viral particles before and after secMFSD6 Mb treatment. Purified EV-D68 treated with secCtl Mb appeared as spherical solid-core particles (full particles) (Fig. 5). Notably, the viral particle morphology changed from solid-core particles to hollow-core particles (empty particles) in response to secMFSD6 Mb treatment, supporting the notion that the viral capsid experiences conformational rearrangements induced by secMFSD6 Mb.

### SecMFSD6 Mb suppresses EV-D68 replication in respiratory cell lines

**Fig. 6:**
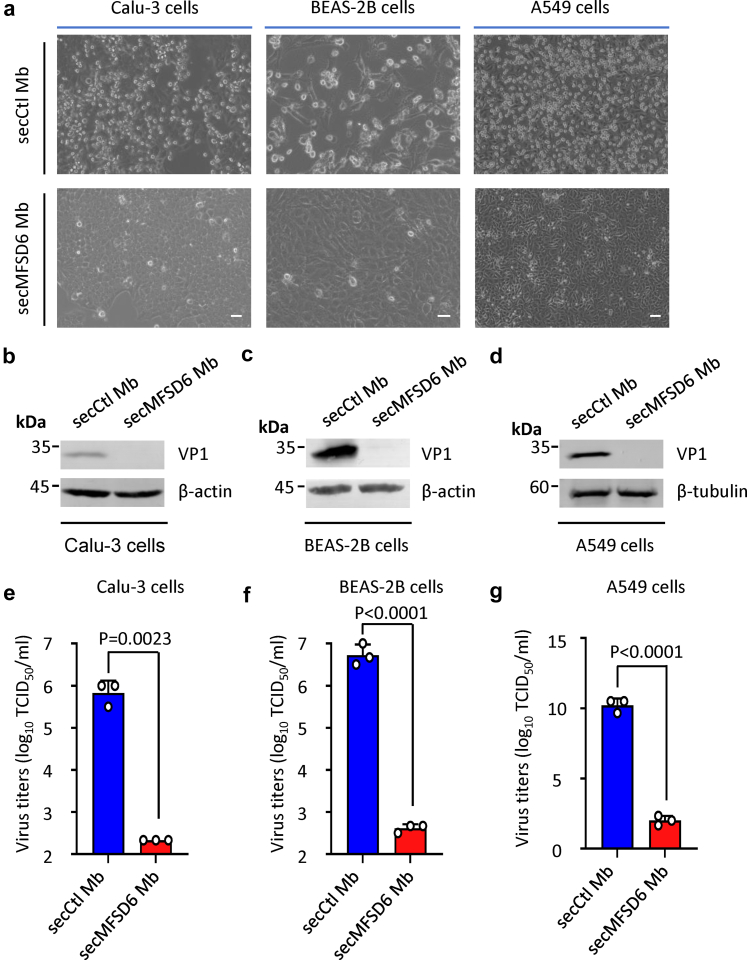
**secMFSD6 Mb inhibits EV-D68 viral replication in respiratory cell lines.** (a–g) EV-D68 MO viruses were incubated with secCtl Mb or secMFSD6 Mb for 30 min. Calu-3 cells, BEAS-2B cells, and A549 cells were infected with treated EV-D68 MO viruses. (a) EV-D68-induced cytopathic effects (CPEs) were inhibited by secMFSD6 Mb. Scale bars equal 20 μm (BEAS-2B cells) or 70 μm (Calu-3 cells and A549 cells). (b–d) The secMFSD6 Mb inhibits viral protein VP1 expression in respiratory cell lines (Calu-3 cells, b; BEAS-2B cells, c; A549 cells, d). (e–g) The secMFSD6 Mb inhibits viral titres in the supernatant of respiratory cell lines (Calu-3 cells, e; BEAS-2B cells, f; A549 cells, g). Data are represented as the mean ± SD (n = 3). *N* = 3 biological replicates. Welch’s t-test (e) or unpaired t-test (f–g) was used to assess statistical significance.

As a respiratory virus, EV-D68 infects and spreads among humans by targeting respiratory cells. Therefore, we further investigated the effects of secMFSD6 Mb intervention on EV-D68 infection in various immortalized human respiratory epithelial cells, including Calu-3, BEAS-2B, and A549. Our results revealed that secMFSD6 Mb treatment efficiently protected against cytopathic effects (CPEs) induced by the EV-D68 MO virus in Calu-3, BEAS-2B, and A549 cells (Fig. 6a), inhibited the accumulation of viral proteins (Fig. 6b–d), and reduced the production of EV-D68 progeny viruses (Fig. 6e–g). Similarly, the replication of another circulating strain KY and the prototype strain Fermon was also inhibited by secMFSD6 Mb in respiratory cells (Supplementary Fig. S3). These results indicate that secMFSD6 Mb has antiviral activities during EV-D68 infection in respiratory cells.

### SecMFSD6 Mb suppresses EV-D68 infection in vitro and in vivo

**Fig. 7:**
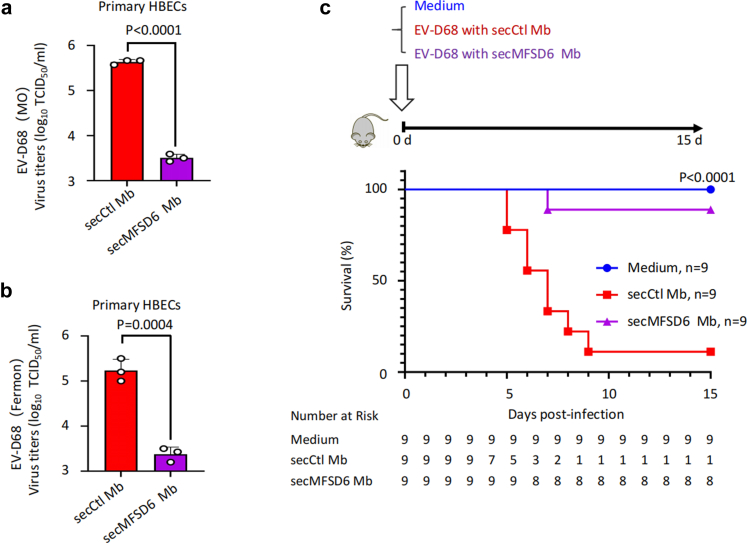
**secMFSD6 Mb inhibits EV-D68 viral replication in vitro and in vivo.** (a and b) EV-D68 viruses were incubated with secCtl Mb or secMFSD6 Mb for 30 min. Primary human bronchial epithelial cells (HBECs) were infected with treated EV-D68 viruses. The secMFSD6 Mb inhibits viral titres in the supernatant (MO, a; Fermon, b). (c) Neonatal ICR mice (n = 9 per group) were intraperitoneally inoculated with EV-D68 US/MO/14-18947 (1.0 × 10^7^ TCID_50_) pre-incubated with secCtl Mb or secMFSD6 Mb and monitored daily for 15 days. The corresponding number of animals at risk for each group throughout the time course is indicated in the lower panel. The log-rank (Mantel–Cox) test was employed in this study. Data are represented as the mean ± SD (n = 3). *N* = 3 biological replicates. Unpaired t-test (a and b) was used to assess statistical significance.

To better assess the potential application of secMFSD6 Mb in combating EV-D68 infection and pathogenesis, we examined its antiviral effects in primary human bronchial epithelial cells (HBECs). As expected, EV-D68 replication was markedly inhibited by secMFSD6 Mb, including both the circulating strain MO (Fig. 7a) and the prototype strain Fermon (Fig. 7b).

Next, we evaluated the in vivo antiviral activity of secMFSD6 Mb using an EV-D68-infected mouse model. Equal amounts of EV-D68 viruses were pre-incubated with either secCtl Mb or secMFSD6 Mb, respectively, and then the virus samples were intraperitoneally injected into newborn ICR mice. After 16 days of observation, we found that secMFSD6 Mb delayed the lethal effects of EV-D68 compared with the control group treated with secCtl Mb, resulting in a higher survival rate (secCtl Mb: 11.11%, secMFSD6 Mb: 88.89%) (Fig. 7c). These results highlight the potential clinical application of this candidate drug against EV-D68 infection.

## Discussion

Given the increasing incidence of EV-D68 and its association with severe respiratory illnesses and neurological complications, there is an urgent need to develop effective therapeutics.^36^, ^37^, ^38^, ^39^, ^40^, ^41^, ^42^, ^43^, ^44^, ^45^ The lack of widely accepted clinical interventions highlights the necessity for innovative strategies to inhibit viral entry and replication. Building on our previous work identifying the viral receptor MFSD6, we have successfully engineered a secreted microbody derived from MFSD6. This secMFSD6 microbody demonstrates highly efficient antiviral activity against EV-D68.

A notable challenge in targeting EV-D68 lies in its utilisation of multiple receptors for host cell entry. While sialic acid, ICAM-5, and heparan sulphate have been implicated in viral adhesion, our recent identification of MFSD6 as a key receptor mediating viral invasion in respiratory cells adds complexity to the receptor landscape.^31^^,^^32^^,^^46^ The coexistence of these receptors suggests that EV-D68 may exploit redundant entry pathways, complicating efforts to block infection via single-receptor targeting. However, our findings reveal that the secMFSD6 Mb, a secreted decoy receptor engineered from the second ectodomain of MFSD6, exhibits inhibitory effects across diverse EV-D68 strains. This efficacy observed in immortalized cell lines, primary respiratory epithelial cells, and murine infection models implies that the receptor-binding epitopes on EV-D68 particles are either highly conserved or exhibit substantial overlap among strains. Such conservation highlights the therapeutic potential of strategies targeting these critical epitopes, whether through decoy receptors or neutralising antibodies.

The antiviral mechanism of the secMFSD6 Mb extends beyond simple receptor competition. In enteroviruses, receptors are categorized into attachment receptors (mediating cell surface adhesion) and uncoating receptors (facilitating viral genome release). Our biochemical and structural analyses demonstrate that the MFSD6 Mb not only occupies receptor-binding sites but also directly destabilizes viral particles. Density gradient centrifugation revealed that MFSD6 Mb treatment redistributes viral RNA into non-particle fractions, while electron microscopy visualised a striking increase in empty viral capsids. These observations suggest that MFSD6 may play a dual role in EV-D68 infection, functioning both as an attachment receptor and a mediator of uncoating. By mimicking this receptor’s interaction with the virus, the MFSD6 Mb induces premature structural disintegration, effectively neutralising viral infectivity in extracellular environments. This dual mechanism represents an advantage over conventional inhibitors that target only receptor binding or post-entry processes.

From a translational perspective, the MFSD6 Mb strategy offers distinct advantages. First, its efficacy against both historical (e.g., Fermon) and contemporary epidemic strains (e.g., 2014 North American isolates) suggests evolutionary conservation of MFSD6 dependency, reducing the likelihood of viral escape. Moreover, the secreted form of MFSD6 Mb simplifies the production process, enabling the acquisition of antiviral substances directly from the culture supernatant of producer cells. This approach minimises production costs while reducing potential losses of Mb activity during cell lysis.

Beyond EV-D68, antiviral development for enteroviruses is constrained by several intertwined challenges. First, most infections are either asymptomatic or manifest as non-specific febrile that overlap with many other pathogens, complicating timely diagnosis and the design of clinical trials with unambiguous efficacy endpoints. Second, enteroviruses replicate rapidly and typically produce acute, self-limiting disease; by the time symptoms appear, viral loads are often already declining, leaving only a narrow therapeutic window. Third, hundreds of enteroviral serotypes have been identified, and rapid subtyping of patients infected with enteroviruses remains clinically impractical. Crucially, different enteroviruses use distinct cellular receptors, making broad-spectrum inhibition via a single viral receptor decoy strategy highly challenging. Instead, future cocktail strategies that combine multiple receptor decoys may offer a promising approach to counter the diversity of enteroviruses.

While our findings demonstrate promising antiviral activity of secMFSD6 Mb, several limitations warrant attention. First, phylogenetic analyses of EV-D68 have revealed marked genetic diversity, with strains classified into genotypes A through D based on VP1 sequences; notably, sub-genotype B3 is currently the most prevalent clade worldwide, followed by A2/D2. However, the EV-D68 strains used in this study (US/MO/14-18947 and US/KY/14-18953, both isolated in the US in 2014) belong exclusively to the B1 clade. Thus, broader testing across phylogenetically diverse contemporary strains is essential to define clinical relevance. Additionally, as these strains were purchased from ATCC and have undergone multiple laboratory passages, potential phenotypic drift from original clinical isolates must be considered.^2^^,^^36^ Second, current efficacy data derive solely from immortalised cell lines and neonatal (1-day-old) ICR mice; studies in more physiologically relevant models, such as humanized mice or non-human primates, are required to better approximate human respiratory infection. Third, critical translational parameters, including pharmacokinetics, immunogenicity, and repeat-dose toxicity, remain uncharacterised. Furthermore, head-to-head comparisons of MFSD6 Mb efficacy with existing neutralising antibodies or capsid binders in human organoids or humanized models are needed. Finally, experimental evidence from serial viral passaging studies is required to assess the theoretical barrier to resistance. These gaps represent priority areas for future investigation.

While the physiological role of MFSD6 remains undefined, our murine studies indicate no overt toxicity from short-term Mb administration. Nevertheless, future investigations into MFSD6’s native functions will guide Mb optimisation—for instance, truncating or modifying its viral-binding domains to eliminate potential cross-reactivity with endogenous MFSD6. Such refinements could further enhance therapeutic specificity without compromising antiviral potency.

In summary, this study establishes the secMFSD6 microbody as a multifaceted antiviral agent capable of neutralising EV-D68 through receptor competition and direct virion destabilization. Its efficacy across in vitro and in vivo models, coupled with scalable production and broad strain coverage, positions this decoy receptor strategy as a promising therapeutic platform. Beyond its immediate implications for EV-D68, this work illustrates the broader potential of decoy receptors in combating rapidly evolving viral pathogens. By exploiting conserved entry mechanisms, such approaches may transcend the limitations of strain-specific countermeasures, offering a durable solution to emerging infectious threats.

## Contributors

Conceptualisation: WW; Study design: WW, JZ, HG; Data collection: ZL, HL, XL, DG, WY, HX; Data analysis: WW, ZY, CD, ZL, HL, XL; Data interpretation: WW, ZY, ZL, HL, XL, DG; Writing-original draft: WW, JZ, HG; Review and editing: WW, JZ, HG, ZY, ZL, HL, XL, DG, CD. WW, ZL, HL, XL accessed and verified the underlying data. All authors have read and approved the final version of this manuscript.

## Data sharing statement

Data collected for the study will be made available and shared with others though contact at the following address: wwei6@jlu.edu.cn.

## Declaration of interests

The authors declare no competing interests.

## Appendix A Supplementary data

Antibody validation

Cell validation

Supplemental Western blots
