# Supplementary material for: Characterisation of a secreted MFSD6-Fc microbody as a decoy receptor for respiratory enterovirus D68
Source: eBioMedicine. 2025 Sep 8;120:105915. doi: 10.1016/j.ebiom.2025.105915 (PMC12452593; doi:10.1016/j.ebiom.2025.105915)
Supplement: Antibody validation [file mmc1.docx]

**Antibody validation**

| Protein name | Catalogue number | Manufacture | RRID |
| --- | --- | --- | --- |
| Rabbit polyclonal anti-enterovirus D68 VP1 | GTX132313 | GeneTex | AB_2886609 |
| Mouse monoclonal anti-HA | H9658 | Sigma-Aldrich | AB_260092 |
| Mouse monoclonal anti-beta Tubulin | M1305-2 | HuaBio | AB_3073058 |
| Beta Actin Mouse Monoclonal Antibody | EM21002 | HuaBio | AB_2819164 |
| Goat polyclonal anti-rabbit IgG (H + L) | 111-035-045 | Jackson ImmunoResearch | AB_2337938 |
| Goat polyclonal anti-mouse IgG (H + L) | 115-055-062 | Jackson ImmunoResearch | AB_2338533 |
