# Supplementary material for: Characterisation of a secreted MFSD6-Fc microbody as a decoy receptor for respiratory enterovirus D68
Source: eBioMedicine. 2025 Sep 8;120:105915. doi: 10.1016/j.ebiom.2025.105915 (PMC12452593; doi:10.1016/j.ebiom.2025.105915)
Supplement: Cell validation [file mmc2.docx]

| Cell line designation | Catalogue number | Manufacture | RRID |
| --- | --- | --- | --- |
| Human: A549 cells | CRM-CCL-185 | ATCC | CVCL_0023 |
| Human: BEAS-2B cells | CRL-3588 | ATCC | CVCL_0168 |
| Human: Calu-3 cells | HTB-55 | ATCC | CVCL_0609 |
| Human: HEK293T cells | CRL-3216 | ATCC | CVCL_0063 |
| Human: RD cells | CCL-136 | ATCC | CVCL_1649 |

**Cell line validation**
