# Supplementary figures and images for: Characterisation of a secreted MFSD6-Fc microbody as a decoy receptor for respiratory enterovirus D68
Source: eBioMedicine. 2025 Sep 8;120:105915. doi: 10.1016/j.ebiom.2025.105915 (PMC12452593; doi:10.1016/j.ebiom.2025.105915)

Figure 2b

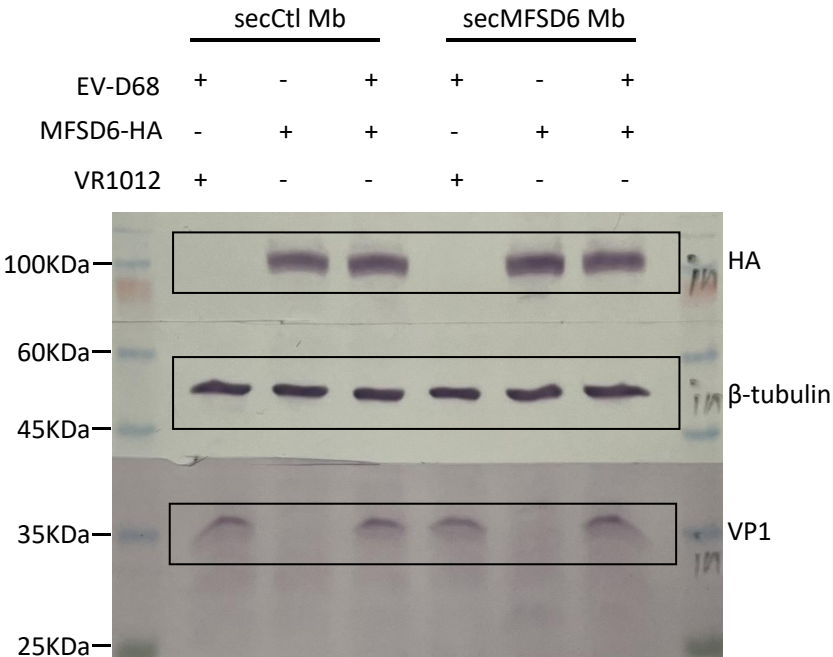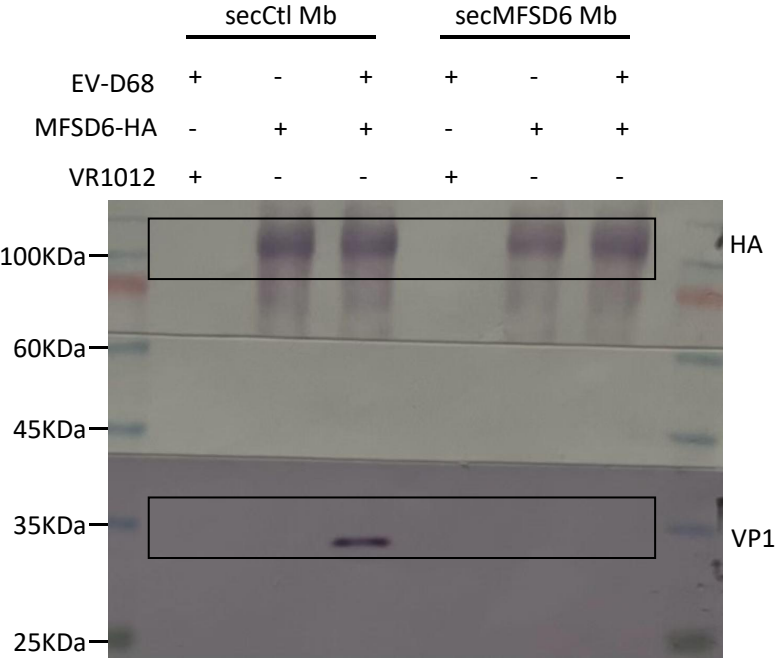

Figure 6b

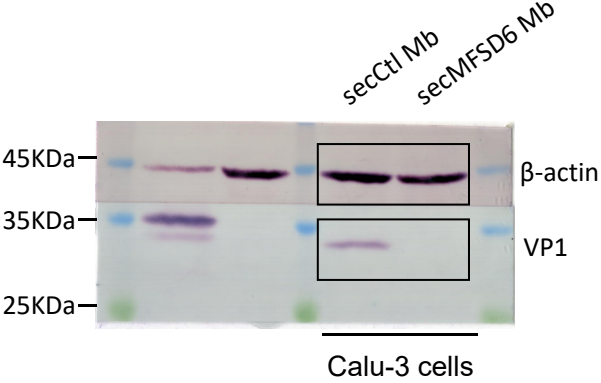

Figure 6c

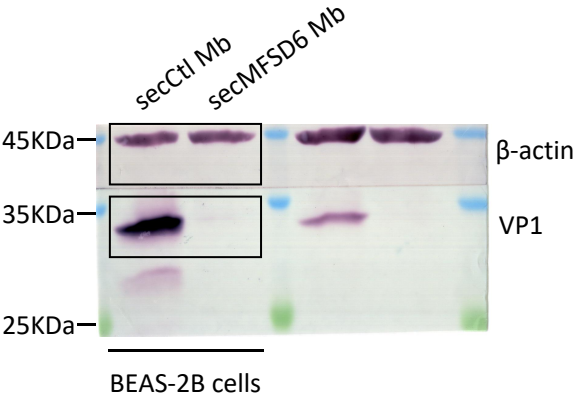

Figure 6d

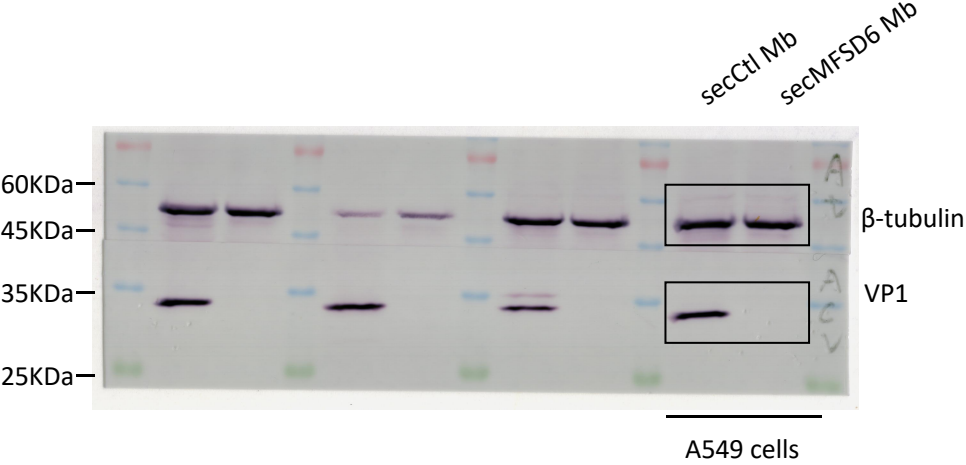

Supplement: Supplemental Western blots [file mmc3.pdf]
