# Supplementary material for: Characterisation of a secreted MFSD6-Fc microbody as a decoy receptor for respiratory enterovirus D68
Source: eBioMedicine. 2025 Sep 8;120:105915. doi: 10.1016/j.ebiom.2025.105915 (PMC12452593; doi:10.1016/j.ebiom.2025.105915)
Supplement: Supplementary Figs. S1–S3 [file mmc4.docx]

**Appendix A Supplementary data**

**Characterisation of a Secreted MFSD6-Fc Microbody as a Decoy Receptor for Respiratory Enterovirus D68**

Zhaoxue Li, Huili Li, Xize Liu, Junfeng Zhou, Delong Gao, Wanying Yang, Huiming Xia, Chao Dou, Zhenglei Yu, Haoran Guo, and Wei Wei*

**
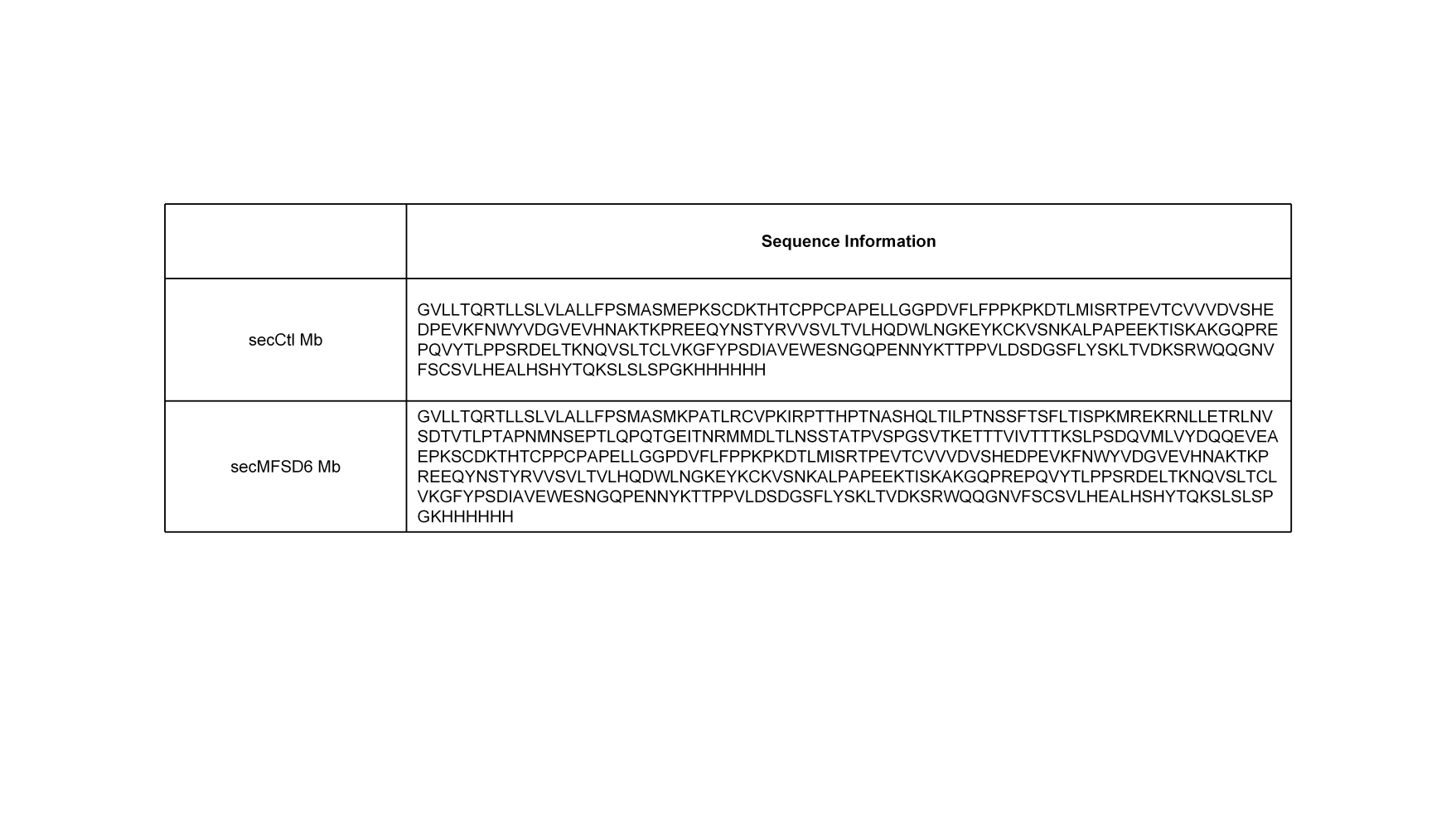
**

**Figure S1. Amino acid sequences of secMFSD6 Mb and secCtl Mb.**

**
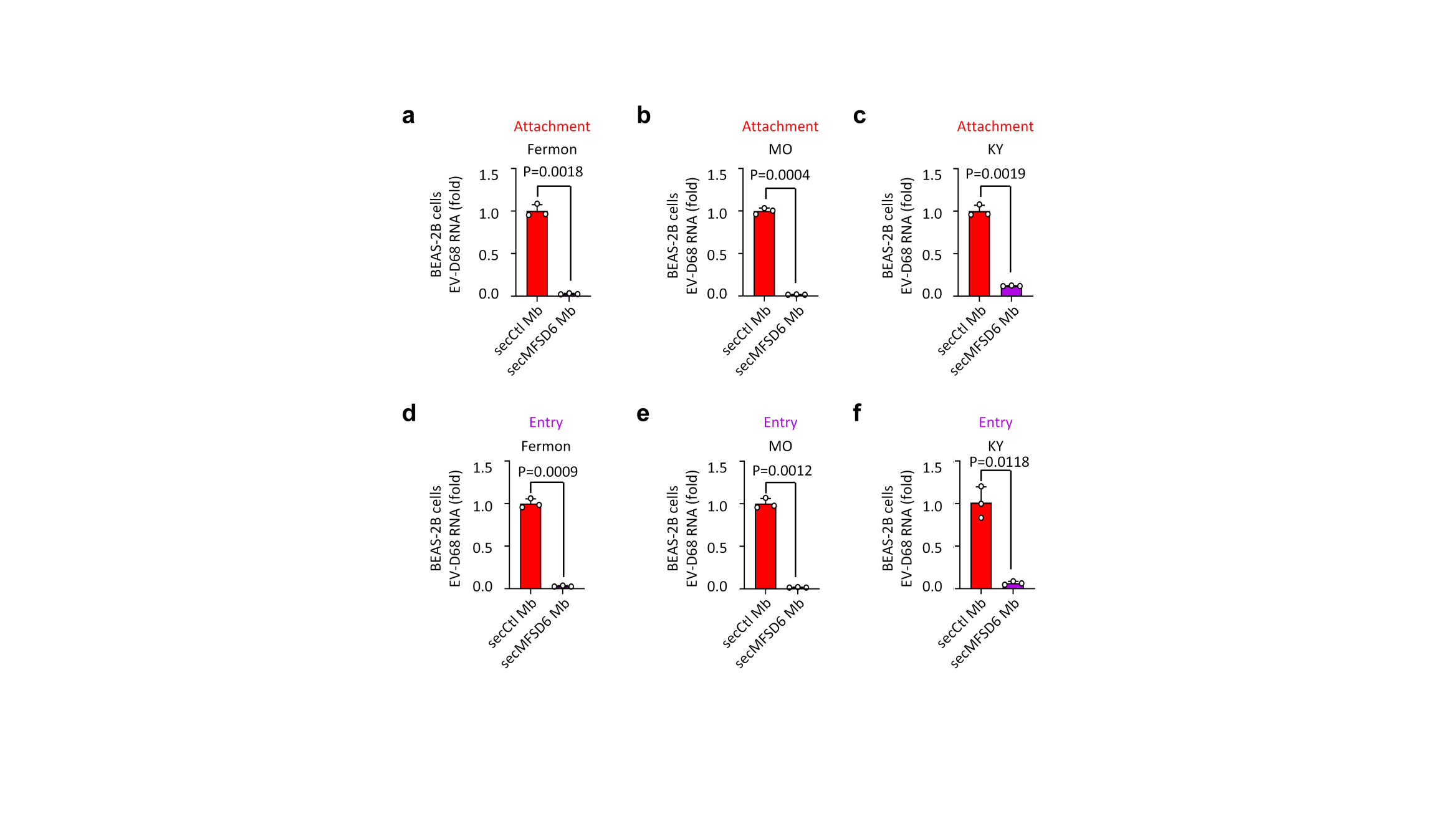
**

**Figure S2. secMFSD6 Mb Blocks EV-D68 Entry into Cells**

(a-f) EV-D68 viruses were incubated with secCtl Mb or secMFSD6 Mb for 30 min.

(a-c) BEAS-2B cells were washed with cold PBS before infection and then incubated with treated EV-D68 (Fermon, a; MO, b; KY, c) or secCtl Mb or secMFSD6 Mb at 4 °C for 2 h to facilitate virus attachment.

(d-f) BEAS-2B cells were washed with prewarmed PBS before infection and then incubated with treated EV-D68 (Fermon, d; MO, e; KY, f) or secCtl Mb or secMFSD6 Mb at 37 °C for 2 h to facilitate virus entry. Data are represented as the mean ± SD (n=3). *N* = 3 biological replicates. Welch’s t-test (a-f) was used to assess statistical significance.


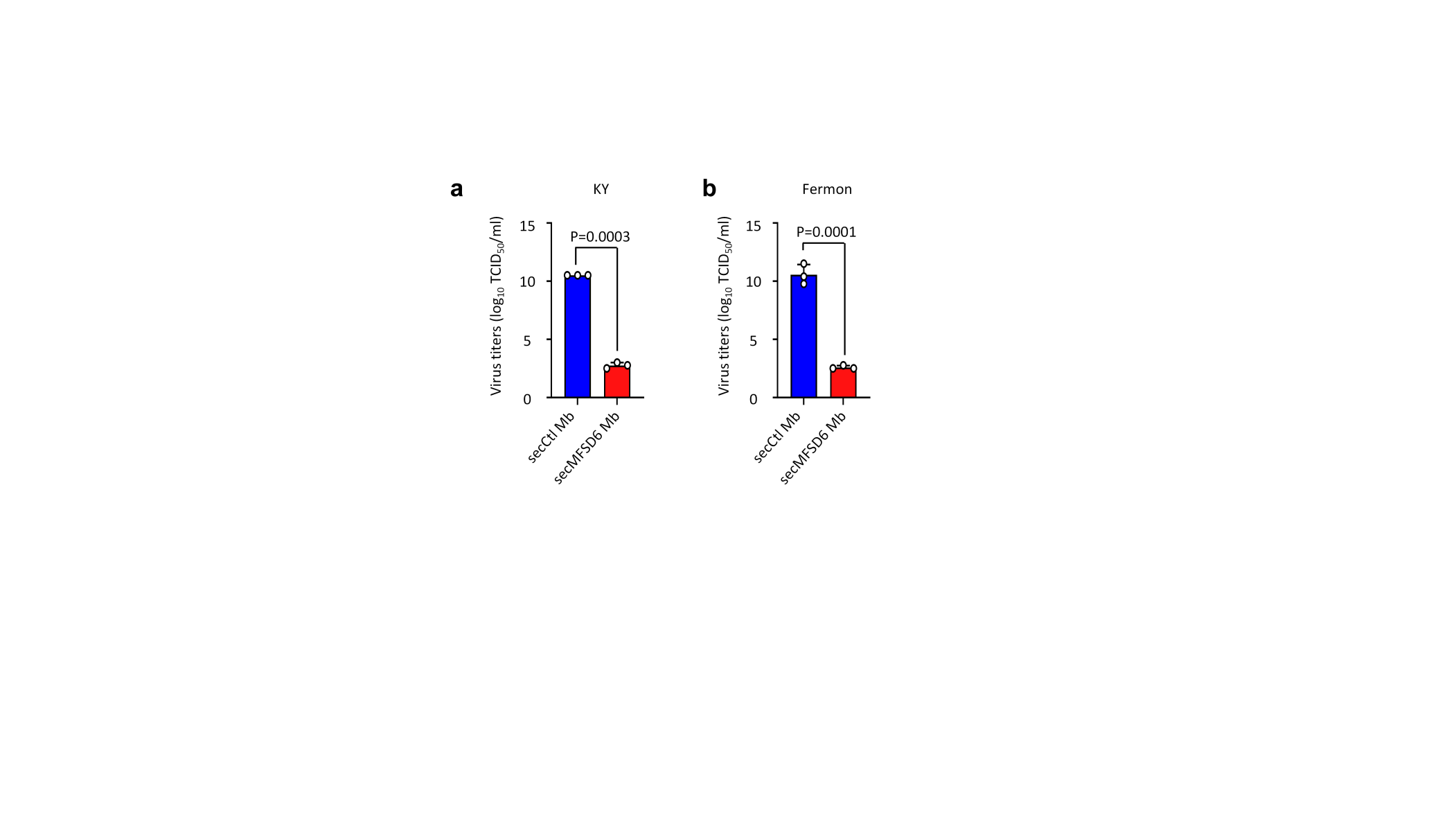


**Figure S3. secMFSD6 Mb Inhibits the Replication of Different EV-D68 Strains**

(a-b) EV-D68 viruses were incubated with secCtl Mb or secMFSD6 Mb for 30 min. A549 cells were infected with treated EV-D68 viruses. The secMFSD6 Mb inhibits viral titers in the supernatant (KY, a; Fermon, b). Data are represented as the mean ± SD (n=3). *N* = 3 biological replicates. Welch’s t-test (a) or unpaired t-test ( b) was used to assess statistical significance.
